# The transhepatic endotoxin gradient is present despite liver cirrhosis and is attenuated after transjugular portosystemic shunt (TIPS).

**DOI:** 10.1186/1471-230X-11-107

**Published:** 2011-10-06

**Authors:** Daniel Benten, Julian Schulze zur Wiesch, Karsten Sydow, Andreas Koops, Peter Buggisch, Rainer H Böger, Charlotte A Gaydos, Helen Won, Veronica Franco, Ansgar W Lohse, Stuart C Ray, Ashwin Balagopal

**Affiliations:** 1 Department of Gastroenterology and Hepatology, University Hospital Hamburg-Eppendorf, Martinistr. 52, 20246 Hamburg, Germany; 2 Department of Interventional Cardiology, University Hospital Hamburg-Eppendorf, Martinistr. 52, 20246 Hamburg, Germany; 3 Department of Diagnostic and Interventional Radiology, University Hospital Hamburg-Eppendorf, Martinistr. 52, 20246 Hamburg, Germany; 4 Institute for Clinical Pharmacology, University Hospital Hamburg-Eppendorf, Martinistr. 52, 20246 Hamburg, Germany; 5 Division of Infectious Diseases, Department of Medicine, The Johns Hopkins Medical Institutions, The Johns Hopkins Medical Institutions, 855 N. Wolfe Street, Baltimore, Maryland, USA

## Abstract

**Background:**

Translocation of gut-derived bacterial products such as endotoxin is a major problem in liver cirrhosis.

**Methods:**

To assess the hepatic clearance of bacterial products in individuals with cirrhosis, we tested concentrations of Gram-negative bacterial lipopolysaccharide (LPS), LPS-binding protein (LBP), and the precursor of nitric oxide (NO), L-arginine, in a cohort of 8 stable patients with liver cirrhosis before and after elective transjugular portosystemic shunt (TIPS) implantation, including central venous, hepatic venous, and portal venous measurements.

**Results:**

Using an adapted LPS assay, we detected high portal venous LPS concentrations (mean 1743 ± 819 pg/mL). High concentrations of LPS were detectable in the central venous blood (931 ± 551 pg/mL), as expected in persons with cirrhosis. The transhepatic LPS gradient was found to be 438 ± 287 pg/mL, and 25 ± 12% of portal LPS was cleared by the cirrhotic liver. After TIPS, central venous LPS concentrations increased in the hepatic and central veins, indicating shunting of LPS with the portal blood through the stent. This paralleled a systemic increase of L-arginine, whereas the NO synthase inhibitor asymmetric dimethylarginine (ADMA) remained unchanged, suggesting that bacterial translocation may contribute to the pathogenesis of circulatory dysfunction post-TIPS.

**Conclusions:**

This study provides quantitative estimates of the role of the liver in the pathophysiology of bacterial translocation. The data indicate that the cirrhotic liver retains the capacity for clearance of bacterial endotoxin from the portal venous blood and that TIPS implantation attenuates this clearance. Thus, increased endotoxin concentrations in the systemic circulation provide a possible link to the increased encephalopathy in TIPS patients.

## Background

Systemic translocation of gut-derived bacteria and their products is a risk factor for recurrent spontaneous bacterial peritonitis and/or encephalopathy in patients with liver cirrhosis [1,2]. For decades, there has been great interest in unraveling the pathophysiology of bacterial translocation in cirrhosis [3]. Experimental data derived from animal studies demonstrated portal and systemic bacteremia, predominantly Gram-negative organisms, in cirrhotic animals with portal hypertension [4-6]. Research conducted 20-30 years ago suggested an important role for endotoxemia in liver cirrhosis, but the assays available at that time were less sensitive and human portal blood sampling is challenging [6,7]. Recent detection of bacterial translocation during HIV infection, thought to result from increased gut permeability, provided a plausible cause of systemic immune activation; however, parallel studies in, HBV, HCV and HIV-HCV co-infection demonstrated that bacterial translocation is strongly associated with cirrhosis, raising questions of cause and effect [8-11]. In this context, we and others have improved the sensitivity of standard LPS assays [12].

The implantation of a transjugular intrahepatic portosystemic shunt (TIPS) has become a standard procedure for the treatment of complications of portal hypertension [13,14]. Although of proven benefit in decompensated liver cirrhosis, TIPS is associated with significant mortality and morbidity from worsened hepatic encephalopathy and hematogenous infections [15]. It is thought that these complications of TIPS are, in part, due to the diminished hepatic clearance of bacterial products [16], although the change in clearance has not been measured. The implantation of TIPS offers an opportunity to directly quantify LPS in various compartments of the human circulation including the portal vein [17,18].

Here, we assess concentrations of LPS and the precursor of nitric oxide (NO), L-arginine, in a cohort of patients with liver cirrhosis undergoing TIPS implantation to determine the transhepatic LPS gradient in vivo before and after TIPS [17-19].

## Methods

### Patients

Plasma samples were collected from 8 consecutive patients with liver cirrhosis and either refractory ascites, or recurrent bleeding from varices, undergoing TIPS placement between 2006 and 2007. Spontaneous bacterial peritonitis was excluded in all patients using ascitic fluid criteria (< 250 polymorphonuclear cells per μL), and no patient had systemic infection or active variceal bleeding. Informed consent was obtained from each patient included in the study and the study protocol conforms to the ethical guidelines of the 1975 Declaration of Helsinki as reflected in *a priori *approval by the institution's human research committee.

### Methods

Blood was obtained during a standard TIPS procedure. After cannulation of the internal jugular vein and insertion of a LEV2 catheter (Cook Medical, Germany), blood was obtained before TIPS from the right atrium (central venous) and from the hepatic vein close to the junction with the inferior vena cava. After puncture of the portal vein, blood was drawn from its trunk. Approximately 15 min after TIPS placement, blood was again obtained from the hepatic vein at the junction to the inferior vena cava. The final blood sample after TIPS placement was again drawn from the right atrium. All blood samples were immediately cooled on ice, centrifuged for 10 min under 2,000 g and plasma was stored at -80°C. All patients were fasting for at least 8 hours before the procedure.

LPS concentrations were measured in plasma diluted to 1:250 and 1:500 using a Limulus Amebocyte Lysate assay (LONZA, Walkersville, MD) as previously described [8], with modifications [12]. For LPS-binding protein (LBP) measurement, plasma was diluted 1:800 and measured using a commercially available plate-based ELISA assay according to the manufacturer's specifications (Cell Sciences, Canton, MA).

For quantification of 16s rDNA, DNA was extracted from a 200 μL aliquot of plasma samples after lysis and purification using the Roche MagNA Pure system (Roche Diagnostics, Indianapolis, IN). 16s rDNA was PCR amplified using a set of universal primers and probe (forward primer [P891F] 5'TGGAGCATGTGGTTTAATTCGA; reverse primer [P1033R] TGCGGGACTTAACCCAACA; UniProbe CACGAGCTGACGACARCCATGCA) that have been validated as sensitive and specific for bacterial detection to the limit of 5 pg of contaminating DNA [20,21].

L-arginine, asymmetric dimethylarginine (ADMA) and symmetric dimethylarginine (SDMA) plasma concentrations were determined by liquid chromatography tandem mass spectrometry as recently described [22].

### Statistical analyses

The paired t-test was used for inter-group comparisons. All data are expressed as mean ± SD. P values of 0.05 or less were considered significant.

## Results

**Figure 1:**
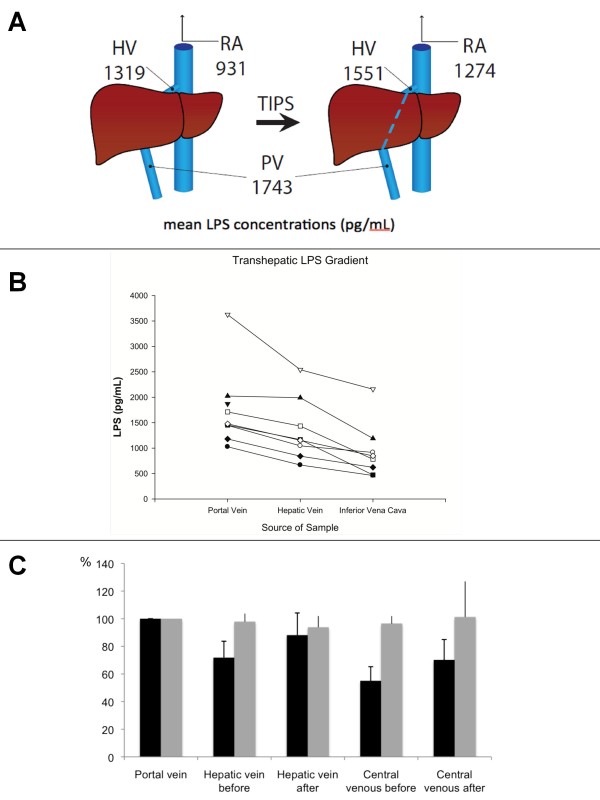
**LPS concentrations before and after TIPS**. **A: **Schematic view of the portal and systemic circulation, showing mean LPS quantities before and after TIPS placement. **B: **Individual LPS concentrations of the portal and systemic circulation and the transhepatic gradient before TIPS (* = statistical significant difference) **C: **Amount of LPS (black bars) and LBP (grey bars) in portal and systemic circulatory compartments shown as percentage of the values in the portal vein, before and after TIPS.

Patient characteristics are shown in Table 1. No patient had active variceal bleeding since endoscopic therapy had been successful weeks ago in the two affected patients. LPS was detectable in the central venous blood of all patients before TIPS, although individual concentrations were highly variable (mean 931 ± 551 pg/ml). Before TIPS placement, the LPS concentrations in the portal vein were higher than in the hepatic vein in all patients (mean 1743 ± 819 vs. 1319 ± 645 pg/ml, p < 0.05), indicating significant clearance of LPS by the cirrhotic liver (Figure 1A-B). Indeed, the mean pre-TIPS transhepatic endotoxin gradient (THEG) of 438 ± 287, which was calculated as endotoxin concentration in the portal vein minus the endotoxin concentration in the hepatic vein, was consistently found in all patients (Figure 1B). TIPS insertion was technically successful in all patients, as demonstrated by fluoroscopy with flow of the portal venous blood through the stent. In blood samples taken 15 min after TIPS placement, LPS concentrations in the hepatic vein were higher than before TIPS in all but one patient (mean 1319 ± 645 vs. 1551 ± 778 pg/ml p < 0.05). Similarly, central venous LPS concentrations after TIPS were higher than before intervention (mean 1274 ± 921 vs. 931 ± 551 pg/ml, p < 0.05) (Figure 1A).

**Table 1:** Patient characteristics

Patient no	Gender/age	Cause of liver cirrhosis	Indication for TIPS	CPS	AST/ALT (U/l)	MELD Score	CRP (mg/dl)	Lactulose treatment	Systemic antibiotics	Central venous LPS before TIPS (μmol/l)
1	f/60	Alcohol	Ascites, hepatorenal syndrome	C	49/32	11	< 5	y	y	461
2	m/47	Alcohol	Ascites	C	83/60	24	< 5	n	n	915
3	m/66	Alcohol	Ascites, variceal bleeding	B	43/35	13	26	y	n	473
4	m/72	Alcohol	Ascites, partial portal vein thrombosis	A	24/14	7	10	n	n	781
5	m/32	PSC	Varices, partial portal vein thrombosis	A	44/56	10	10	n	n	622
6	f/48	Alcohol	Ascites	B	47/14	17	10	y	n	847
7	f/40	Alcohol	Ascites	B	50/15	7	6	n	n	1190
8	m/59	Alcohol	Recurrent variceal bleeding, hydrothorax	C	36/16	13	12	y	y	2158

Abbreviations: no, number; f, female; m, male; PSC, Primary sclerosing cholangitis; CPS, Child-Pugh Score Class; CRP, c-reactive protein; y, yes; n, no

To facilitate comparison of individual clearance rates pre-/post-TIPS insertion in the various compartments, LPS concentrations were individually normalized to levels in the portal vein (set as 100% in each patient, Figure 1C). On average, the pre-TIPS LPS concentration in the hepatic vein was 75 ± 12%, which corresponds to a hepatic LPS clearance of 25 ± 12%. After TIPS, the hepatic vein LPS concentration increased to 92 ± 16% of the portal vein concentrations, a trend indicating shunting of LPS with the portal venous blood through the stent and loss of the THEG. The central venous LPS concentration increased from 52 ± 10% to 68 ± 15% of the portal venous concentration (Figure 1C).

LBP is intimately connected with the host response to LPS, but freely circulates in all blood compartments and is not expected to be cleared by the liver; therefore, as a negative control we measured LBP concentrations in the right atrium, hepatic, and portal vein and found no transhepatic gradient before or after TIPS (Figure 1C). Bacterial DNA was measured using a quantitative PCR assay for 16s rDNA. Bacterial DNA was found in 37/40 tested samples, but there was no relationship or gradient between 16s rDNA concentrations pre- and post-TIPS in the various blood compartments (data not shown).

It has recently been shown that the presence of bacterial DNA, a marker of bacterial translocation, was associated with aggravation of NO-mediated peripheral vasodilation in patients with liver cirrhosis [19]. In addition NO production resulted from endotoxemia [23]. Thus, we measured L-arginine as an NO precursor in the various compartments before and after TIPS. In the portal vein, L-arginine plasma concentrations were higher than in the hepatic vein before TIPS (77.4 ± 10.0 vs. 51.2 ± 8.1 μmol/l, p < 0.01). After TIPS, concentrations in the hepatic vein increased to 77.0 ± 11.8 μmol/l, similar to the portal vein levels. Also, the central venous L-arginine concentration increased from 66.9 ± 12.4 μmol/l pre-TIPS to 78.4 ± 13.2 μmol/l after TIPS (p < 0.05). Interestingly, concentrations of the endogenous NO synthase inhibitor ADMA and SDMA were similar in all compartments before and after TIPS (p = n.s., data not shown).

## Discussion

In healthy individuals, only minimal amounts of endotoxin are found in peripheral venous blood, since hepatic macrophages (Kupffer cells) are believed to clear the endotoxin from intestinal bacteria that enter the portal vein [3,24-26]. In contrast, endotoxin is frequently detected in the peripheral blood of cirrhotic patients resulting from both increased translocation from the gut and reduced hepatic clearance [27-29]. However, clinical studies on the pathophysiology of bacterial translocation and hepatic endotoxin clearance in cirrhosis have been challenging due to the problem of accessing the portal vein in humans, emphasizing the demand for new diagnostic strategies. Recently, a trial in cirrhotic patients has shown that presence of bacterial DNA, a marker of bacterial translocation, was associated with aggravation of peripheral vasodilation and worsening of intrahepatic endothelial dysfunction [19]. To further assess the impact of bacterial translocation in patients with decompensated cirrhosis, we screened a series of patients undergoing elective TIPS implantation for changes in endotoxemia before and after shunting blood from the liver.

A striking difference from previous studies is the high concentration of systemic endotoxemia detected in our cohort of patients. The *Limulus *Amebocyte Lysate (LAL) assay for LPS quantification has been extensively reported to be inhibited by mammalian plasma and serum. We have recently reported that in a cohort of HIV-infected persons and SIV-infected macaques, plasma dilutions of ≥ 1:200 were required to diminish the assay inhibition of plasma, revealing concentrations comparable with those reported here [12].

Recently, a well-designed trial in patients undergoing emergency TIPS for uncontrollable variceal bleeding has examined hemodynamic changes in systemic and cerebral blood flow and found systemic endotoxemia to be associated with increased NO production [18]. The assessment of systemic LPS concentrations in such severely ill patients, however, may not be representative of our cohort, which consists of stable patients receiving elective TIPS given the strong possibility of transient endotoxinemia due to instrumentation and bleeding in the former group [30,31]. Paradoxically, only one-half of individuals in that study had detectable LPS and even post-TIPS values were comparatively low [18], which may be explained by the practice of broad-spectrum antibiotic administration in persons with acute variceal bleeding.

We found that the cirrhotic liver cleared 25% of the endotoxin present in the portal vein. The proportion of LPS that we measured in the central vein compared to the portal vein (52 ± 10%) compared closely with a previous analysis from Lumsden et al. 1988, who determined that 57% of portal LPS was cleared compared to the systemic circulation [27]. In contrast, we analyzed for the first time the THEG between the portal vein and the hepatic vein directly pre- and post TIPS, which better reflects hepatic clearance due to absence of dilution from the systemic venous circulation. The THEG pre-TIPS was found to be 438 ± 287 pg/mL, and was remarkably similar between patients despite variable portal and hepatic vein LPS concentrations in the cohort. The pre-TIPS THEG we observed strongly indicates that the liver retains the capacity for portal blood detoxification even in the setting of cirrhosis, but this may be an underestimate of LPS clearance because cirrhosis is frequently associated with reversal of flow, spontaneous portosystemic shunting, and impaired clearance of bacteremia. Ideally it would be interesting to assess the same values in control patients without liver cirrhosis. However, this is practically challenging, since access to portal venous and hepatic venous blood is ethically justified only in persons with a clinical indication for TIPS placement.

LBP concentrations were not differentially enriched in the various blood compartments, suggesting that the transhepatic LPS gradient was not simply an artifact due to progressive hepatic vein dilution. Since LBP -in contrast to LPS- is not specifically cleared by Kupffer cells, LBP amounts were expected to be the same in the portal and hepatic veins. LBP is produced by hepatocytes, and circulating quantities may be diminished with extensive hepatic fibrosis and cirrhosis. Reduced LBP levels may impair LPS sensing systemically, since it is a key component of the TLR4 receptor complex. The negative immunologic consequences of reduced LPS sensing in the context of impaired LPS clearance potentially includes both poor innate immune surveillance with resulting bacterial infections, as well as reduced immunotolerance resulting in sepsis-like syndromes; both are known complications of end-stage liver disease.

Bacterial DNA concentrations were elevated in all patients, but the levels were not different in the hepatic and portal vein, suggesting that clearance of bacterial DNA and LPS occurs via different mechanisms. Markers of impaired intestinal permeability are elevated in portal hypertension, particularly with severe liver disease, and in viral and alcoholic cirrhosis [11,32]. Circulating LPS levels have indeed been associated with extensive hepatic fibrosis in HCV and HBV infection [8,11]. Therefore, although we observed an increase in hepatic venous and systemic endotoxemia in the short term, this may be reversed in the follow-up period after TIPS placement, since portal hypertension is reduced. In accordance, three studies observed reduction of surgical infections or peritonitis after lowering portal venous pressures [33-35]. Indeed, it is important to highlight that despite the patients' clinical status as having decompensated cirrhosis, we found that the liver retains the capacity to clear translocated bacterial products and that a decrease in this capacity post-TIPS implantation is associated with increased NO concentrations. Since no long-term peripheral endotoxin concentrations are available in our cohort of patients, future studies should focus on the potential improvement of endotoxemia and the incidence of infections after TIPS placement.

Endogenous NO is important for the integrity of the intestinal mucosa, whereas overproduction of NO, e.g. by inducible NO synthase (iNOS), impairs function of the intestinal epithelium, increases its permeability and leads to bacterial translocation and inflammatory responses [36,37]. Further complicating inference of cause and effect, bacterial translocation and endotoxemia increase NO production in animal models of cirrhosis and in patients [23,38,39]. In accordance with a recent study focusing on hepatic clearance of NO synthase inhibitors [40], we found the highest values for L-arginine in the portal venous blood. Also, L-arginine concentrations in the hepatic vein and central venous blood increased after TIPS to a level as high as in the portal vein. In contrast, the endogenous NOS inhibitor ADMA was unaltered before and after TIPS. This increase in NO bioavailability after TIPS placement may be one of the factors contributing to the hyperdynamic state after TIPS placement. Whether this results solely from shunting through the TIPS or is a consequence of reduced cleareance of endotoxin by the liver remains unclear. It has been suggested that arginase-1 expressing, alternatively activated macrophages regulate collagen production and organ fibrosis [41]. Whether the increased L-arginine, which serves as a substrate for arginase-1, is also involved in alteration of liver fibrosis is still unclear and needs to be studied in animal models [42]. However, studies in cirrhotic patients demonstrated that intestinal decontamination with antibiotics is associated with less systemic endotoxemia and less NO-mediated vasodilatation [23,43], thus indicating a potential link to the observed changes after TIPS placement. Since intestinal decontamination has recently shown to improve encephalopathy and prognosis in cirrhotic patients [44,45], future studies should focus on the underlying pathophysiology including analysis of portal/systemic endotoxin and NO physiology in patients with cirrhosis. LPS concentrations should be correlated with clinical parameters and outcome, and clinical interventions should be tested to lower endotoxemia in patients undergoing TIPS, i.e. by application of prophylactic periinterventional or long-term antibiotics.

## Conclusions

Taken together, this small pilot study demonstrates for the first time a significant transhepatic gradient of LPS and L-arginine in vivo, and immediate loss of this gradient following TIPS. These findings also suggest that measurements of microbial translocation from peripheral blood are likely to be both underestimates of intestinal translocation and significantly confounded by liver disease. The liver retains some capacity for LPS clearance even in cirrhosis, and bypassing hepatic LPS may increase the risk of infection after TIPS. In addition, future studies of microbial translocation must include assessment of liver function, particularly in studies of HIV-infected people in whom liver disease is a major cause of death [46].

## Competing interests

The authors declare that they have no competing interests.

## Authors' contributions

DB designed the study, acquired TIPS samples, analyzed data, wrote and revised the manuscript. JSzW designed the study, analyzed data, wrote and revised the manuscript. KS measured and interpreted NO-related data and revised the manuscript. AK and PB performed TIPS and acquired samples. RHB developed NO-related assays and interpreted data. CAG, HW and VF analyzed bacterial DNA samples. VF performed the LPS and LBP assays. AWL participated in study design and revised the manuscript. SCR and AB designed the study concept, measured LPS/LBP and participated in writing the manuscript.

All authors read and approved the final manuscript.

## Pre-publication history

The pre-publication history for this paper can be accessed here:

http://www.biomedcentral.com/1471-230X/11/107/prepub
